# Effects of whole-body neuromuscular electrical stimulation device on hemodynamics, arrhythmia, and sublingual microcirculation

**DOI:** 10.1007/s00380-020-01755-1

**Published:** 2021-02-06

**Authors:** Megumi Hoshiai, Kaori Ochiai, Yuma Tamura, Tomoki Tsurumi, Masato Terashima, Hajime Tamiya, Eikou Maeno, Satoshi Mizuguchi, Takashi Tomoe, Atsuhiko Kawabe, Atsuko Uema, Asuka Ueno, Takushi Sugiyama, Yasuto Horie, Hiroyuki Sugimura, Ryousuke Koike, Takanori Yasu

**Affiliations:** 1 Department of Cardiovascular Medicine and Nephrology, Dokkyo Medical University Nikko Medical Center, Nikko, Tochigi Japan; 2 Department of Rehabilitation, Dokkyo Medical University Nikko Medical Center, Nikko, Tochigi Japan; 3 Department of Cardiology, Dokkyo Medical University Nikko Medical Center, Nikko, Tochigi Japan; 4 Department of Pulmonology, Dokkyo Medical University Nikko Medical Center, Nikko, Tochigi Japan

**Keywords:** Arrhythmia, Oxidative stress, Rheology, Whole-body neuromuscular electrical stimulation, Microcirculation

## Abstract

Neuromuscular electrical stimulation has been used to treat cardiovascular diseases and other types of muscular dysfunction. A novel whole-body neuromuscular electrical stimulation (WB-NMES) wearable device may be beneficial when combined with voluntary exercises. This study aimed to investigate the safety and effects of the WB-NMES on hemodynamics, arrhythmia, and sublingual microcirculation. The study included 19 healthy Japanese volunteers, aged 22–33 years, who were not using any medication. Electrocardiogram (ECG), echocardiography, and blood sampling were conducted before a 20-min WB-NMES session and at 0 and 10 min after termination of WB-NMES. Their tolerable maximum intensity was recorded using numeric rating scale. Arrhythmia was not detected during neuromuscular electrical stimulation or during 10 min of recovery. Blood pressure, heart rate, left ventricular ejection fraction, and diastolic function remained unchanged; however, mild mitral regurgitation was transiently observed during WB-NMES in a single male participant. A decrease in blood glucose and an increase in blood lactate levels were observed, but no changes in blood fluidity, sublingual microcirculation, blood levels of noradrenaline, or oxidative stress were shown. WB-NMES is safe and effective for decreasing blood glucose and increasing blood lactate levels without changing the blood fluidity or microcirculation in healthy people.

## Introduction

Exercise therapy is an inexpensive, safe, and extremely effective primary and secondary intervention for the prevention of cardiovascular and chronic respiratory diseases. Reduced exercise capacity after primary percutaneous coronary intervention in acute myocardial infarction patients may lead to worse clinical outcomes [1]. A major issue for rehabilitation in people with restricted movement is how to suppress skeletal muscle atrophy and the decreases in motor function [2, 3]. It was suggested that the start date of gait is related to one of the predictors of activities of daily living in elderly people with heart failure with preserved ejection fraction [4]. Electrical muscle stimulation to encourage skeletal muscle contraction has been a known treatment for disuse-induced muscle atrophy. Even in patients diagnosed with New York Heart Association (NYHA) class IV heart failure, for whom exercise therapy is contraindicated, electrical stimulation can be safely used without affecting blood pressure or heart rate while increasing maximum muscle strength [5]. However, it is known to have important precautions and contraindications. For example, endocrine organs such as the thyroid and genitalia, sensory organs such as the eyes, and people with pacemakers are contraindicated for electrical muscle stimulation [5].

Several studies have used belt-electrode-type skeletal muscle electrical stimulation with electrodes attached to the torso, thighs, and lower legs to examine its effectiveness on circulatory dynamics, muscle contraction and hypertrophy, and safety in patients with arterial disease and diabetes mellitus [6–9]. Nowadays, there are novel and useful wearable devices for whole-body neuromuscular electrical stimulation (WB-NMES), rather than the traditional belt type, that efficiently provide electrical stimulation to the forearms and back arms, chest, back, abdomen, oblique abdomen, gluteus muscles, and front and back thigh muscles [10]. Watanabe et al. [10] have shown that a combination of aerobic exercise and WB-NMES for 15 min enhances metabolic response to the same extent as high-intensity exercise. It would be useful if this exercise regimen could be applied to patients with heart failure who cannot do vigorous exercise. However, the effect of battery-powered WB-NMES devices on hemodynamics, including of the heart and peripheral microcirculation, have not been fully elucidated. Blood fluidity plays an important role in the pathogenesis of various disease and is also a powerful predictor of cardiovascular events [11]. The factors behind the decrease in blood fluidity due to exercise are the transient increase in hematocrit, and the activation of leukocytes and platelets due to catecholamines and cytokines [12]. Strenuous exercise can lead to platelet activation, and hypercoagulation, which can lead to acute coronary syndrome [13]. We have reported that vigorous exercise induced transient deterioration in microcirculation [14]. There is an urgent need to evaluate the safety and effects of electrical stimulation to the whole body, including the chest, on circulatory dynamics, arrythmia, microcirculation, systemic metabolism including blood glucose, lactate levels, and oxidative stress.

This study aimed to examine the safety of a novel WB-NMES wearable device as a modified phase 1 study and its effect on circulatory dynamics, arrythmia, sublingual microcirculation, blood glucose and lactate levels, and oxidative stress. This will help us evaluate whether it is safe to apply electricity using WB-NMES to the whole body including the chest.

## Materials and methods

### Study participants

The study participants comprised 19 healthy volunteers; non-smoking and non-obese Japanese men (*n* = 10) and women (*n* = 9) aged 22–33 years. All were normotensive with body mass indices of 21–24 kg/m^2^. No abnormalities were found during routine physical examinations and standard laboratory analyses after overnight fasting. Persons taking any medications were excluded. The study protocol was approved by the Ethical Committee of Dokkyo Medical University’s Nikko Medical Center (Approval Number: Nikko 29012). All participants provided written informed consent for participation in this study. All procedures performed in this study complied with the national ethical guidelines for medical and health research involving human participants and with the 1964 Helsinki Declaration and its later amendments or comparable ethical standards. Baseline characteristics of the study participants regarding the routine physical examination and standard laboratory test results are shown in Table 1.

**Table 1:** Baseline haracteristics of the study participants

Characteristics	Total (*n* = 19)
Age (y)	29 ± 4
Male (%)	10 (52%)
Height (cm)	164.6 ± 7.2
Weight (kg)	61.6 ± 10.4
BMI (kg/m^2^)	22.7 ± 3.5
White blood cells (/µL)	6257.1 ± 1665.3
Hematocrit (%)	44.0 ± 3.1
Platelets (× 10^3^/µL)	25.9 ± 4
HDL-C (mg/dL)	57.3 ± 15
LDL-C (mg/dL)	109.4 ± 31.1
Triglycerides (mg/dL)	97.8 ± 45.4
Serum creatinine (mg/dL)	0.7 ± 0.2
Plasma glucose (mg/dL)	84.4 ± 5.8
HbA1c level (%)	5.4 ± 0.2

Data are expressed as the mean ± SD (standard deviation)

*BMI* body mass index, *HDL-C* high-density lipoprotein cholesterol, *LDL-C* low-density lipoprotein cholesterol, *HbA1c* hemoglobin A1c

### Whole blood rheology

We used a microchannel flow analyzer system (BWA-MCFAN, Kikuchi Microtechnology Co., Ltd., Ibaraki, Japan) equipped with a microchannel array chip. Blood was drawn through BK 7-7-4.5 microchannels (Kikuchi Microtechnology Co., Ltd.) as an ex vivo model of microvessels to assess the whole blood rheology and leukocyte activity as previously described [15–17].Within 10 min of collecting blood into heparinized tubes, 0.1 mL of blood was drawn through BK 7-7-4.5 microchannels as an ex vivo capillary model (7854-parallel, 7 × 4.5-μm equivalent cross-section, 30 μm long) under a constant vacuum of 20 cm H_2_O (1.96 kPa). The time required for saline to pass through the microchannels was determined before each blood measurement for calibration. The microscopic motion images of blood passing through the microchannels were monitored and stored on a computer system.

### Sublingual microcirculation

Sublingual microcirculation images were observed using a handheld dark-field CytoCam video microscope (Braedius Medical, Huizen, The Netherlands) [18] before and after the WB-NMES. The total blood vessel density, perfused vessel density, and microcirculatory flow index were assessed before and after the WB-NMES. Total blood vessel density was a measure of the total length of vessel divided by the total surface of the analyzed area. Perfused vessel density was the vessel density times the proportion of perfused vessels. The microcirculatory flow index was determined by dividing the average value after the microcirculation image into four quadrants and assigning a number according to the predominant flow type (0 = no flow, 1 = intermittent, 2 = sluggish, 3 = continuous) [18].

### Derivatives of reactive oxygen metabolites and biological antioxidant potential

Plasma levels of derivatives of reactive oxygen metabolites (d-ROMs) and biological antioxidant potential (BAP) were assessed using a d-ROMs test kit (Diacron, Grosseto, Italy) and BAP test (Diacron), respectively [19, 20]. The d-ROMs test depends on Fenton-like reactions, leading to the formation of lipid peroxy- and alkoxy-radicals that in turn react with a chromogenic substrate. The BAP test quantifies the body’s reducing power to iron, which acts as a sensitive antioxidant.

### Blood lactate levels

The blood lactate concentration was measured with the lactate oxidase method using an automated analyzer (Lactate Pro 2, Arkray, Kyoto, Japan) [21]. For this, 5 ml of blood was obtained from the forearm vein before and after the WB-NMES.

### Echocardiography

Echocardiographic examination was performed with a Philips HD 15 Pure Wave machine (Philips Medical System, USA) equipped with 2.5–3.5 MHz transducers. The left ventricular wall motion, left ventricular ejection fraction, left ventricular diastolic function (E/A, E/E'), 2D color Doppler imaging to detect mitral regurgitation and/or tricuspid regurgitation, and inferior vena cava diameter were assessed by echocardiography before and after WB-NMES. Echocardiographic monitoring was continued during WB-NMES.

### Study protocol

**Fig. 1:**
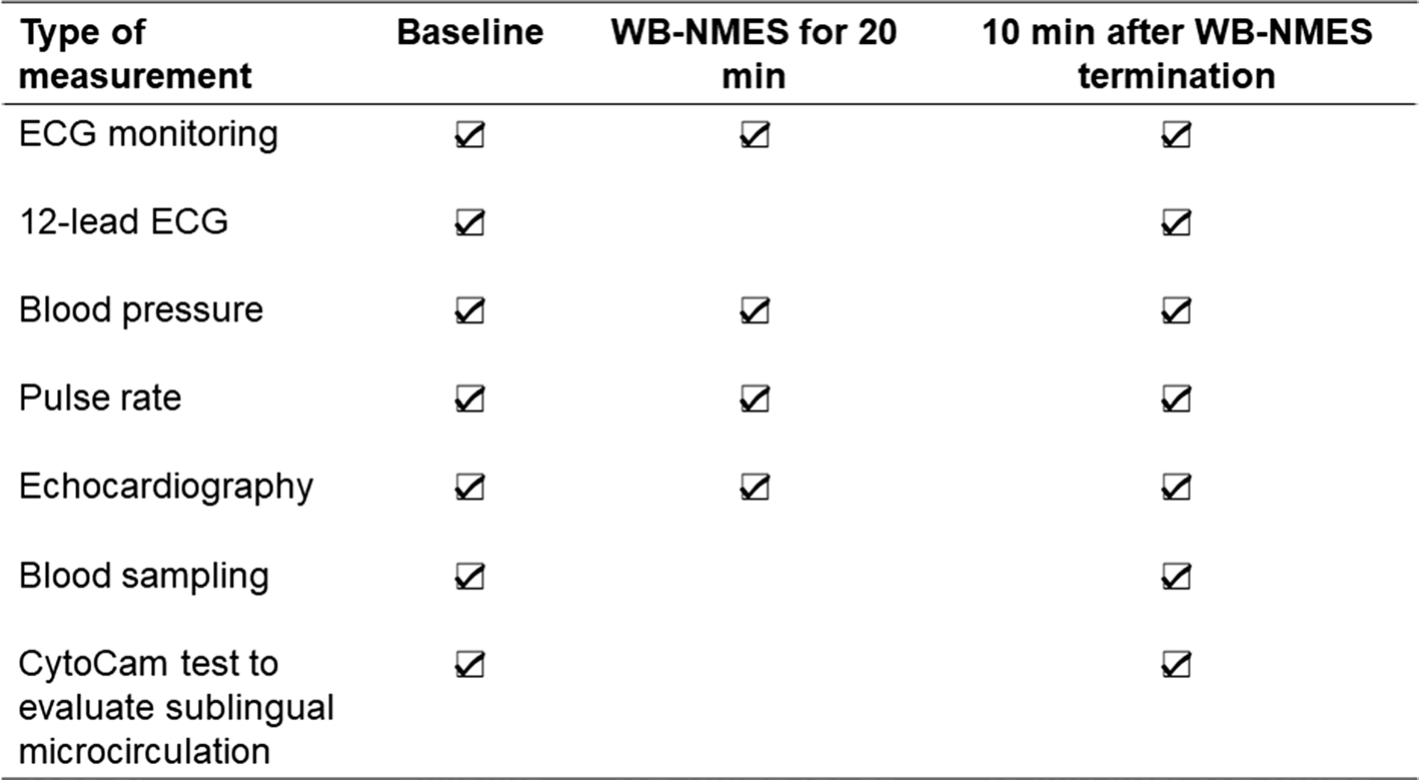
Experimental protocol schedule. 12-lead electrocardiogram (ECG), Blood pressure, pulse rate, echocardiography, blood sampling and CytoCam test were performed on all participants before the whole body neuromuscular electrical stimulation (WB-NMES). ECG monitoring was continued during the study. Echocardiography confirmed wall motion and new appearance of valve during WB-NMES. A 12-lead ECG, echocardiography, blood sampling and CytoCam test were repeated 10 min after termination of the WB-NMES

**Fig. 2:**
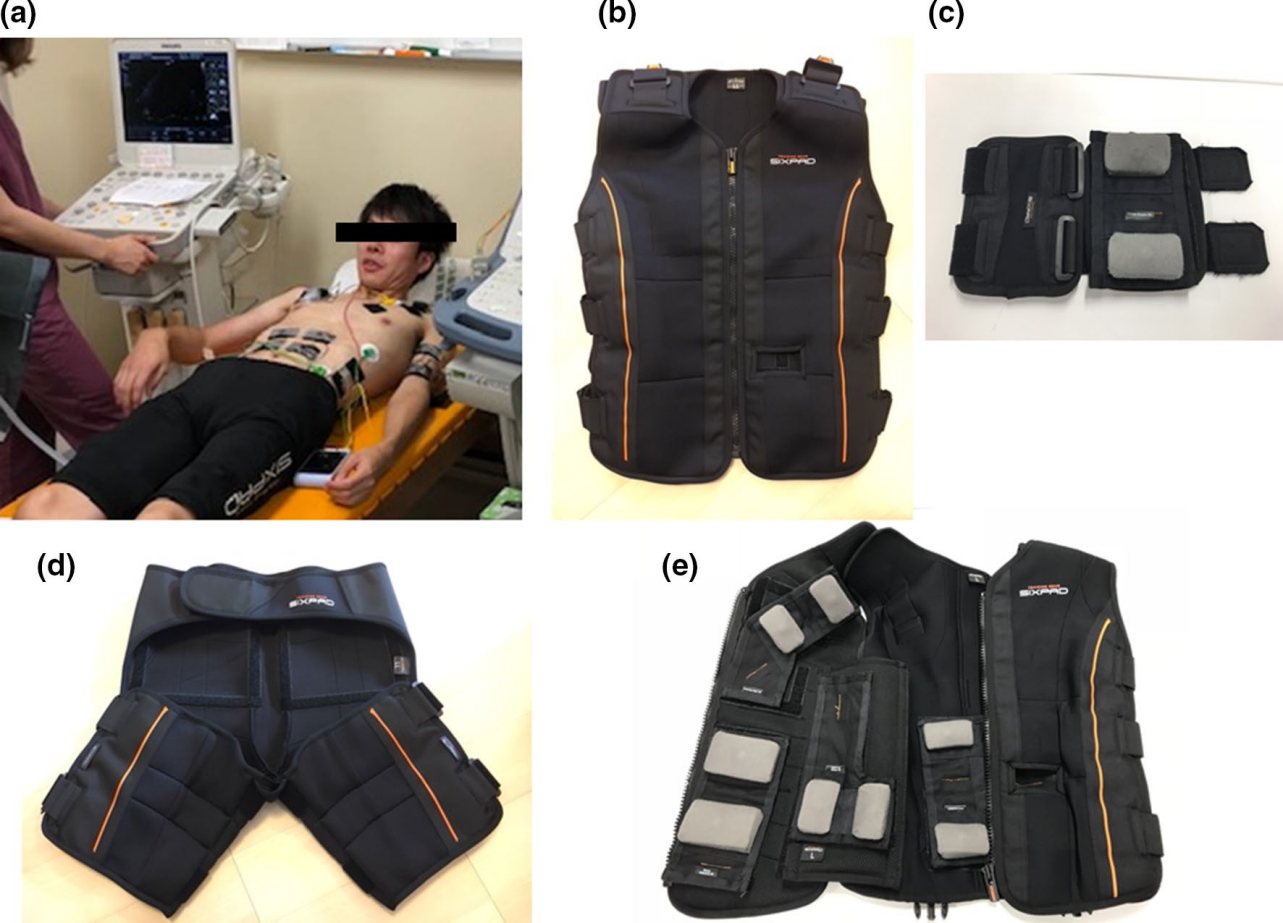
**a** Only the electrical stimulation pads of whole-body neuromuscular electrical stimulation (WB-NMES) are attached. **b** Vest for WB-NMES. **c** Arm band for WB-NMES. The position of the electrodes is shown inside the arm band. In anterior and posterior upper arm. **d** Shorts for WB-NMES. **e** The position of the electrodes is shown inside the vest. In chest, back, abdominal and abdominal oblique

Participants fasted for 5 h and abstained from drinking beverages containing alcohol or caffeine for ≥ 12 h before the study. All experiments were performed in a quiet, temperature-controlled room (23–24 °C) on the same day. The volunteers were instructed to be in supine position. All the tests were supervised by a medical doctor. Echocardiography and 12-lead electrocardiogram (ECG) were performed on all participants before the WB-NMES (Fig. 1). Blood pressure and pulse rate were noninvasively measured every two min (HEM-6130-E, Omron Healthcare Co, Ltd, Kyoto, Japan). ECG monitoring (FCP-8800, Fukuda Denshi Co, Ltd, Tokyo, Japan) was continued during the study. Immediately after blood sampling and assessment of echocardiography, WB-NMES was applied for 20 min to the anterior and posterior upper arm, chest, back, abdominal, abdominal oblique, gluteus, and anterior and posterior thigh muscles using the custom-made stimulator based on a plot type battery-powered WB-NMES device (SIXPAD, MTG Ltd., Nagoya, Japan) (Fig. 2). Electrical stimulation with WB-NMES device was performed for 20 min, replicate the design of a previous study [22]. WB-NMES was performed with the maximum tolerable intensity (Numeric Rating Scale (NRS) > 7) for measurement of discomfort during the treatment. NRS was measured by the participants’ self-report. The protocol for electrical stimulation was performed as previously described in detail [10]. Echocardiography confirmed wall motion and new appearance of a valve during WB-NMES. A 12-lead ECG, echocardiography, and blood sampling were repeated 10 min after termination of the WB-NMES (Fig. 1).

### Statistical analysis

Data are presented as means ± standard deviation for continuous variables and as numbers and percentages for categorical variables. Statistical comparisons were conducted using the Student’s *t*-test, repeated ANOVA, chi-squared test, and Fisher’s exact test. Data were statistically analyzed using JMP 14.0 J software (SAS Institute, Cary, NC, USA). The significance of a two-tailed *P*-value was set at < 5%.

## Results

No arrhythmia was detected during the WB-NMES or after the 10 min recovery period. Blood pressure and heart rate remained unchanged during the WB-NMES and recovery period. Echocardiography showed that the left ventricular ejection fraction and diastolic function were maintained in all participants, but one showed transient mild mitral regurgitation during WB-NMES which lasted a few minutes after termination of WB-NMES (Table 2). Five of the 19 patients received electrical stimulation three times a week for 1 month, with no adverse effects observed.

**Table 2:** Effects of whole-body neuromuscular electrical stimulation on hemodynamics, arrythmia, and echocardiographic parameters

	Baseline (*n* = 19)	During WB-NMES (*n* = 19)	10 min After WB-NMES (*n* = 19)	*P*-value
Arrhythmia on ECG monitor (%)	0	0	0	–
Systolic blood pressure (mmHg)	115.1 ± 10.8	–	119.3 ± 10.4	0.06^a^
Diastolic blood pressure (mmHg)	72.6 ± 9.6	–	71.8 ± 9.6	0.6^b^
Heart rate (beats per min)	71.4 ± 9.7	–	73.2 ± 7.4	0.3^a^
LVEF (%)	60.3 ± 2.8	60.2 ± 2.9	61.0 ± 2.7	0.4^c^
E/A ratio	1.7 ± 0.2	1.6 ± 0.2	1.6 ± 0.2	0.3^d^
Mean E/e’	6.1 ± 1.6	6.3 ± 1.4	6.1 ± 1.2	0.9^d^
IVC (cm)	0.8 ± 0.3	0.6 ± 0.3	0.8 ± 0.2	0.07^d^
Occurrence of TR (%)	0	0	0	–
Occurrence of MR (%)	0	1 (5.3)	0	–

Data are expressed as the mean ± SD, or number (%)

^a^Student’s *t*-test

^b^Wilcoxon signed rank test

^c^One-way repeated measures analysis of variance

^d^Friedman test

*A* atrial systolic wave, *E* early diastolic wave, *e’* early diastolic wall motion velocity, *ECG* electrocardiogram, *IVC* inferior vena cava, *LVEF* left ventricular ejection fraction, *MR* mitral regurgitation, *TR* tricuspid regurgitation

**Fig. 3:**
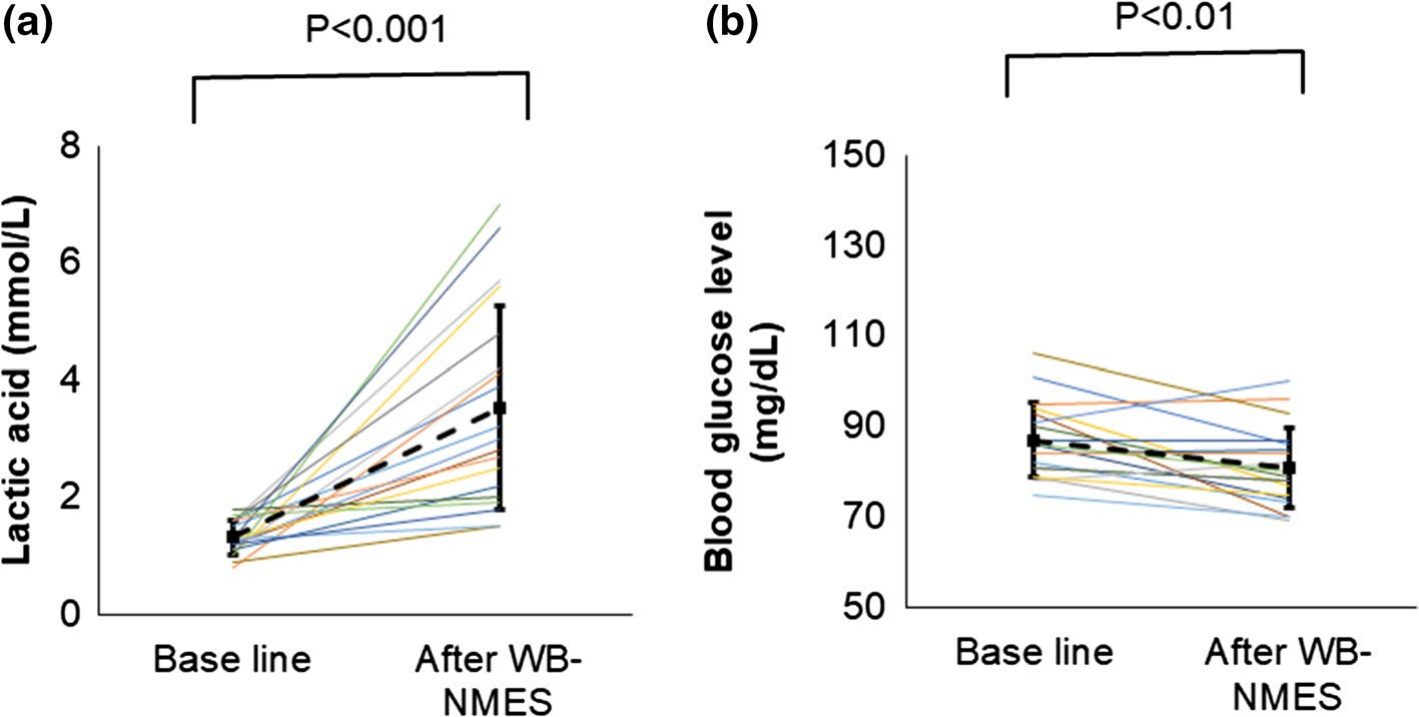
Effects of whole-body neuromuscular electrical stimulation (WB-NMES) on blood level of lactic acid (**a**) and glucose (**b)** shows that WB-NMES significantly increased blood level of lactic acid (**a**) and significantly decreased blood glucose levels (**b**)

**Fig. 4:**
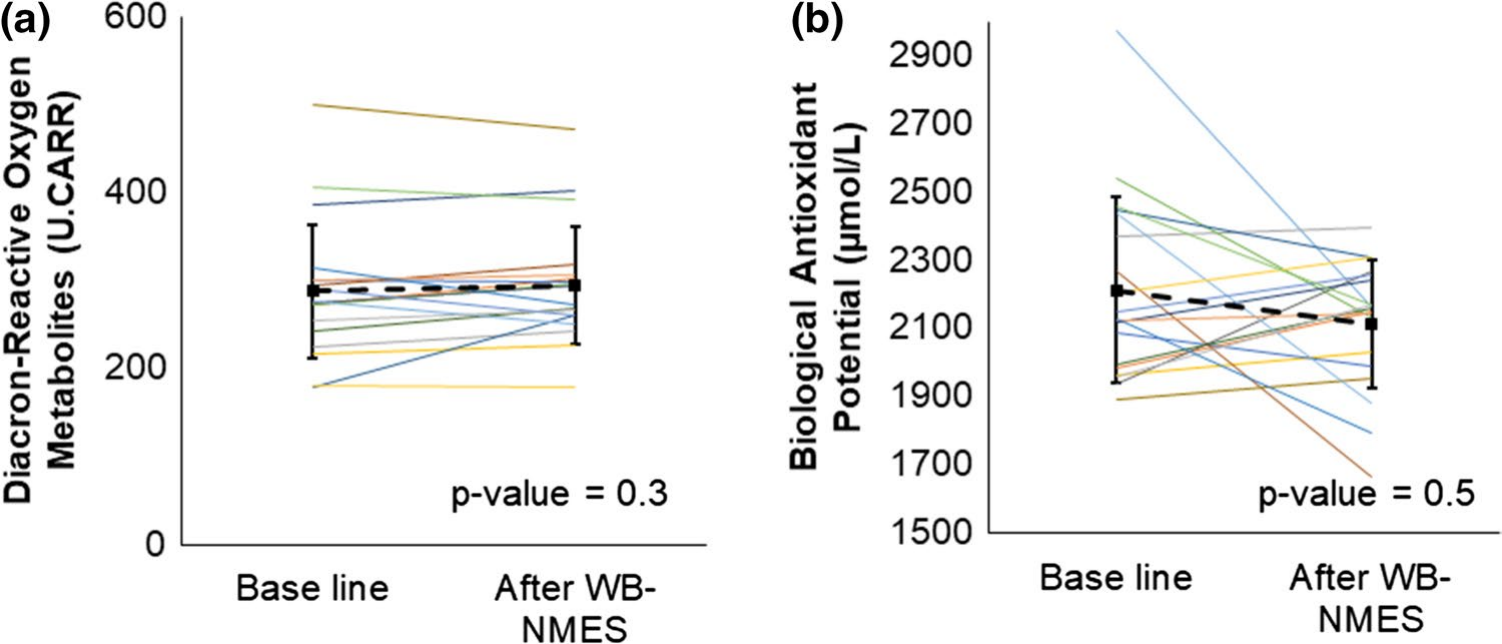
Effects of whole-body neuromuscular electrical stimulation (WB-NMES) on blood level of diacron-reactive oxygen metabolites (**a**) and biological antioxidant potential (**b**) as antioxidant power. The blood level of diacron-reactive oxygen metabolites (**a**) and biological antioxidant potential (**b**) remains unchanged by WB-NMES

**Fig. 5:**
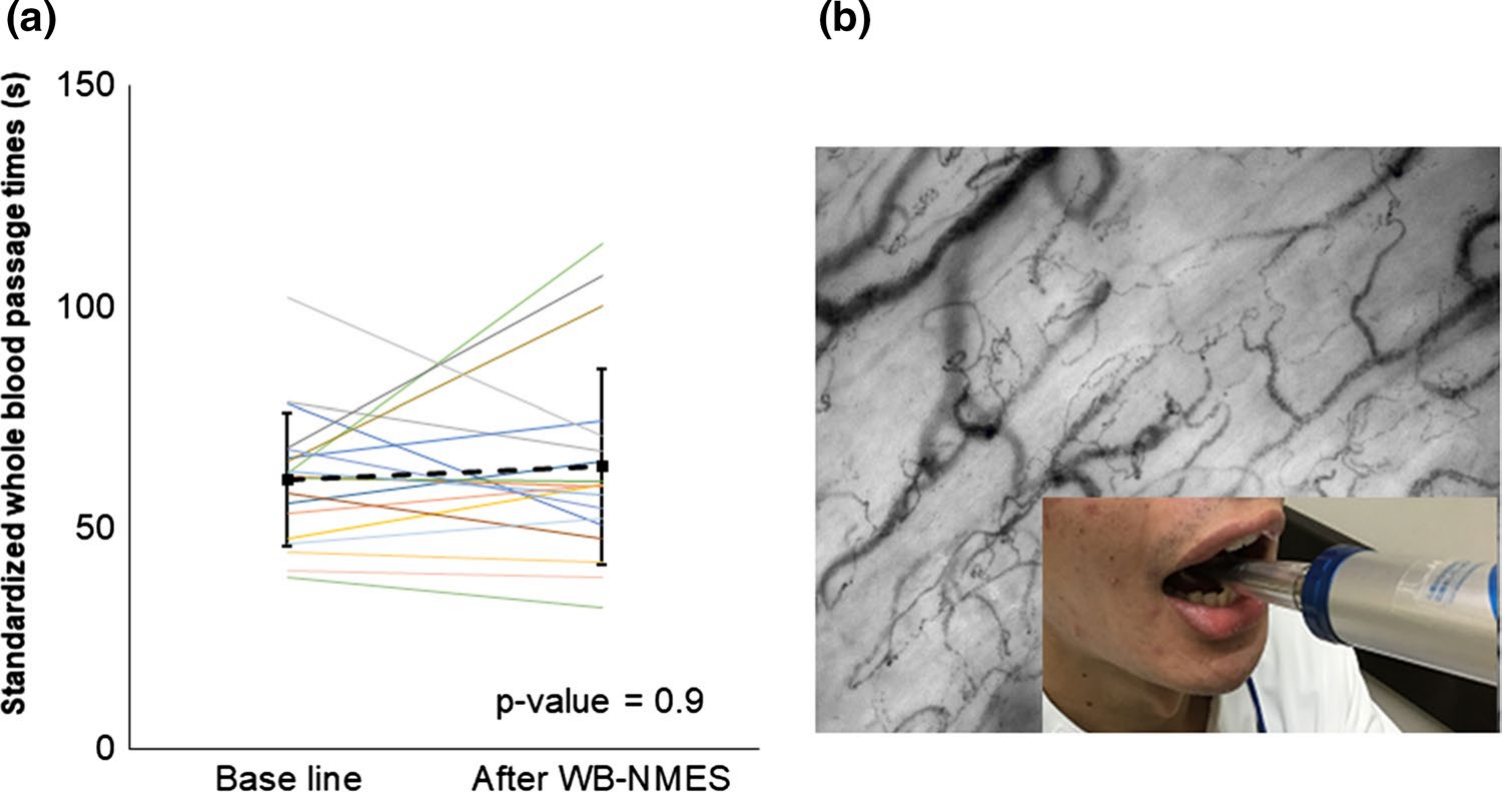
**a** Effects of whole-body neuromuscular electrical stimulation on whole blood passage time as hemorheology and sublingual microcirculation parameters. Whole blood passage time remains unchanged by whole-body neuromuscular electrical stimulation (WB-NMES). **b** A sublingual microcirculation image was captured using a handheld dark-field CytoCam video microscope before and after WB-NMES

Significant decreases in blood glucose and increases in lactate levels were observed (Fig. 3). Although there were no significant differences in d-ROMs, BAP, or blood fluidity (Table 3, Fig. 4), three out of 19 participants showed prolonged whole blood passage time just after WB-NMES. The whole blood passage time of these three participants recovered to baseline measures 20 min after WB-NMES (Fig. 5). The sublingual microcirculation parameters were not statistically different before and after WB-NMES (Table 3, Fig. 5).

**Table 3:** Effects of whole-body neuromuscular electrical stimulation on blood parameters and whole blood passage time

	Baseline (*n* = 19)	10 min after WB-NMES (*n* = 19)	*P*-value
Lactic acid (mmol/L)	1.3 ± 0.3	3.5 ± 1.8	0.00004^a^
Blood glucose level (mg/dL)	87.0 ± 8.1	80.8 ± 9.0	0.004^a^
Noradrenaline (pg/mL)	0.2 ± 0.1	0.2 ± 0.1	0.4^a^
Hematocrit (%)	43.5 ± 3.2	43.1 ± 3.7	0.4^b^
Diacron-reactive oxygen metabolites (U.CARR)	287.2 ± 76.7	294.7 ± 66.6	0.3^b^
Biological antioxidant potential (μmol/L)	2212.2 ± 271.2	2114.4 ± 188.6	0.5^b^
Whole blood passage time (s)	60.6 ± 15.4	64.5 ± 22.6	0.9^b^
Sublingual total blood vessel density (mm/mm^2^)	10.4 ± 2.2	10.3 ± 2.9	0.8^a^
Sublingual perfused vessel density (mm/mm^2^)	6.9 ± 2.0	6.1 ± 3.1	0.3^b^
Sublingual microcirculatory flow index	2.6 ± 0.3	2.5 ± 0.8	0.2^b^

Data are expressed as the mean ± SD (standard deviation), or number (%)

^a^Student’s *t*-test

^b^Wilcoxon signed rank test

*U.CARR* Carratelli unit, 1 U.CARR = 0.08 mg H_2_O_2_/dL

## Discussion

This study aimed to investigate the safety and effectiveness of WB-NMES on hemodynamics, arrhythmia, and sublingual microcirculation. Our results from healthy participants showed that no arrhythmia was detected, while the WB-NMES effectively increased blood lactate level, decreased blood glucose level, and maintained blood pressure, blood fluidity, and sublingual microcirculation.

WB-NMES has been introduced as an alternative to physical training or in combination with low-grade exercise. However, associated muscular damage, as evident by elevation of serum creatine-kinase activity, has been reported [23, 24]. Until more evidence regarding the physiological contraindications is gathered [8, 24], the electrical strength should be prescribed, the health of the users should be considered, and instructors should be trained to avoid WB-NMES-related side effects [25].

Although the efficacy and safety of WB-NMES have been examined, specific guidelines regarding cardiopulmonary function are lacking. A study by Jee et al. [26] reported that there were no abnormal changes in heart rate, systolic and diastolic blood pressure, and oxygen uptake during or after WB-NMES. The major findings of the present study suggest that the application of the maximum tolerable WB-NMES intensity did not cause arrhythmias and did not negatively affect heart rate, systolic and diastolic blood pressure, or cardiac function in healthy young people as observed by echocardiology. Since electrical stimulation is applied near the heart in WB-NMES, it is necessary to confirm its effects on heart function in addition to heart rate, blood pressure, and oxygen intake. In this study, we confirmed the safety of WB-NMES in healthy participants by evaluating the cardiac function with echocardiography. We found transient mild mitral regurgitation in one male subject during WB-NMES. The volume of transient functional mitral regurgitation was too small to measure precisely. Vena contracta at the suction was 1.0 mm with colour area trace method. And his cardiac function was within the normal range. The possible mechanism may be a temporary increase in venous perfusion due to WB-NMES, because the mitral regurgitation was observed during lower extremity NMES without pulmonary hypertension. Lower extremity NMES increases venous return in normal participants and patients with heart disease. The activation of the muscular-venous pump by NMES-induced muscle contraction increases venous return [27]. For this reason, patients with NYHA class IV heart failure (left ventricular ejection fraction < 25%), severe cardiac arrhythmias, and electronic devices such as pacemakers and implanted defibrillators, are listed as contraindications in WB- NMES trials [24–26]. Exercise-induced pulmonary hypertension in patients with secondary mitral regurgitation is a powerful predictor of poor outcomes, with increased risk for cardiac-related death [28]. Therefore, the safety and effects of WB-NMES in patients with heart failure is a target for future research.

Blood glucose was significantly decreased by WB-NMES in this study. This is similar to findings from a previous study [6] that found insulin-independent glucose uptake during muscle contraction when NMES was performed on the lower legs. NMES to the lower legs has been reported to significantly enhance whole-body glucose uptake compared to voluntary ergometry exercise at the identical oxygen uptake in the presence of significantly higher blood lactate and respiratory quotients [6]. The reduction in glucose after NEMS is most likely due to insulin-independent glucose transporter type 4 upload [29]. Although electric stimulation was applied to several regions of the body in the present study, it was not clear whether it had a stronger hypoglycemic effect than general NMES devices. However, it is known that NMES can enhance anaerobic energy metabolism due to the recruitment of high-threshold motor units or muscle fibers associated with glucose metabolism [6, 30].

Lactate accumulates during anaerobic exercise and can be utilized as an energy source by skeletal muscles during voluntary exercise [31]. Lactic acid increased from 1.3 ± 0.3 mmol/L at rest to 3.5 ± 1.8 mmol/L after WB-NMES. In the present study, it was not clear if the maximum effect was reached by applying electrical stimulation over a wider range compared to the lower leg alone. In our pilot study, three participants showed 2.96 ± 0.3 metabolic equivalents (METs) on the cardiopulmonary exercise test during WB-NMES alone. In a previous study in which NMES was applied to the lower leg, the exercise intensity showed 3.0 ± 0.47 METs [32], which is similar to our results. Therefore, these observations suggest that lactate production from skeletal muscle is limited during walking equivalent to three METs, whereas is much higher with WB-NMES at three METs. In addition, aerobic exercise and resistance training increased blood pressure and heart rate, while WB-NMES alone did not increase either. Therefore, we posit that WB-NMES can provide the systemic skeletal muscle a relatively high-intensity exercise load that produces lactate without affecting cardiac function. In addition, Watanabe et al. [10] have reported that combining WB-NMES with low-intensity voluntary exercise lead greater energy expenditure with the metabolic equivalent of 5.31 ± 0.89 Mets and serum lactic acid level of 7.5 mmol/L. They concluded that more lactic acid was produced by combining voluntary exercise and WB-NMES than WB-NMES alone [10].

Blood fluidity was not significantly different before and after WB-NMES in the present study. Several studies have reported that acute exercise at the anaerobic threshold reduces blood fluidity [14, 33]. The decrease is explained by the increased blood viscosity due to the increase of hematocrit induced by dehydration and activation of white blood cells [14], platelets, and cytokine [13]. However, vigorous exercise leads to increased oxidative stress [34], which may be associated with a decrease in insulin secretory ability and aggravates diabetes [35]. An increase in oxidative stress contributes to the development of various diseases such as cancer, atherosclerosis, and aging [36]. The present results showed that d-ROMs and BAP remained unchanged by WB-NMES.

There were limitations to the study to consider. First, the sample size was limited, because the present study was a single-center observational study analysis without control, and thus, causation could not be established. However, the number of 19 participants may be appropriate as a modified phase 1 study. Second, the study participants were limited to healthy young men and women, so the results cannot be applied to other populations. Third, as we gave only one 20-min session of electrical stimulation, we cannot comment on the safety and efficacy of its long-term use.

## Conclusion

WB-NMES applied to healthy young individuals with the tolerable maximum intensity for 20 min significantly decreased blood glucose and increased blood lactate level; however, it did not change hemodynamic parameters, and it did not induce arrythmia or changes in blood fluidity and sublingual microcirculation. The effects of combining resistant training and/or stretching with WB-NMES should be clarified in future studies. In addition, safety and efficacy of WB-NMES in patients with chronic heart disease or chronic respiratory failure should be examined.

## Data Availability

The deidentified participant data will not be shared.
